# Decreasing Histology Turnaround Time Through Stepwise Innovation and Capacity Building in Rwanda

**DOI:** 10.1200/JGO.17.00081

**Published:** 2017-11-16

**Authors:** Gaspard Muvugabigwi, Irenee Nshimiyimana, Lauren Greenberg, Emmanuel Hakizimana, Deo Ruhangaza, Origene Benewe, Kiran Bhai, James R. Pepoon, Alexandra E. Fehr, Paul H. Park, John Butonzi, Cyprien Shyirambere, Alexis Manirakiza, Christian Rusangwa, Danny Milner, Tharcisse Mpunga, Lawrence N. Shulman

**Affiliations:** **Gaspard Muvugabigwi**, **Irenee Nshimiyimana**, **Emmanuel Hakizimana**, **Deo Ruhangaza**, **Origene Benewe**, **John Butonzi**, and **Tharcisse Mpunga**, Butaro District Hospital, Ministry of Health, Butaro; **Alexandra E. Fehr**, **Paul H. Park**, **Cyprien Shyirambere**, **Alexis Manirakiza**, and **Christian Rusangwa**, Partners In Health/Inshuti Mu Buzima, Kigali, Rwanda; **Lauren Greenberg** and **Kiran Bhai**, Partners In Health; **James R. Pepoon**, Brigham and Women’s Hospital, Boston, MA; **Danny Milner**, American Society for Clinical Pathology, Chicago, IL; and **Lawrence N. Shulman**, Abramson Cancer Center, University of Pennsylvania, Philadelphia, PA.

## Abstract

**Purpose:**

Minimal turnaround time for pathology results is crucial for highest-quality patient care in all settings, especially in low- and middle-income countries, where rural populations may have limited access to health care.

**Methods:**

We retrospectively determined the turnaround times (TATs) for anatomic pathology specimens, comparing three different modes of operation that occurred throughout the development and implementation of our pathology laboratory at the Butaro Cancer Center of Excellence in Rwanda. Before opening this laboratory, TAT was measured in months because of inconsistent laboratory operations and a paucity of in-country pathologists.

**Results:**

We analyzed 2,514 individual patient samples across the three modes of study. Diagnostic mode 1 (samples sent out of the country for analysis) had the highest median TAT, with an overall time of 30 days (interquartile range [IQR], 22 to 43 days). For diagnostic mode 2 (static image telepathology), the median TAT was 14 days (IQR, 7 to 27 days), and for diagnostic mode 3 (onsite expert diagnosis), it was 5 days (IQR, 2 to 9 days).

**Conclusion:**

Our results demonstrate that telepathology is a significant improvement over external expert review and can greatly assist sites in improving their TATs until pathologists are on site.

## INTRODUCTION

A staggering fact that has become apparent to all sectors (including funders) is that, as of 2012, deaths resulting from cancer worldwide had exceeded those resulting from HIV/AIDS, tuberculosis, and malaria combined.^1^ Current reports of cancer incidence, prevalence, morbidity, and mortality demonstrate that the largest burden of this disease is found in low- and middle-income countries (LMICs), particularly in sub-Saharan Africa. In a 2011 WHO report, projections for global cancer incidence and mortality between now and 2030 show increases of 65% (85% in Africa) and 71%, respectively, with more than 70% of this total burden falling on LMICs.^2,3^ In spite of this, only 5% of global oncology resources are spent in LMICs.^4,5^

**Fig 1:**
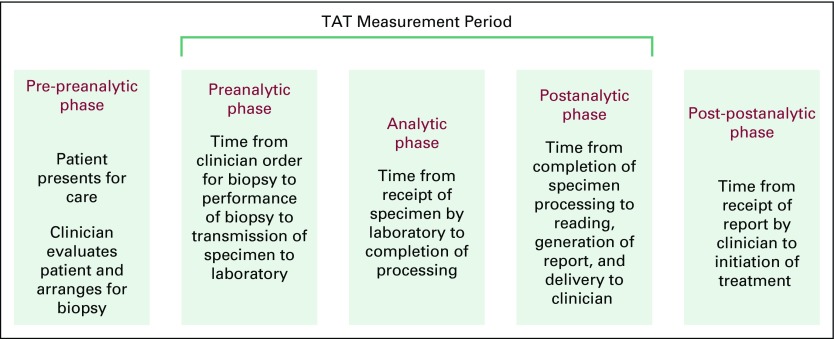
Phases of clinical care and pathology processing and reporting. There are five phases encompassing patient care and ranging from obtaining and processing the specimen to delivering the report to initiating treatment. Although all are crucial, our analysis of turnaround time (TAT) focused on the time from biopsy to the creation of a pathology report and the effect of different diagnostic methods on this TAT.

A pathologic diagnosis is a critical requirement to treat any patient with cancer, regardless of geography or economy.^1,6^ Without an accurate diagnosis, treatment plans that will optimize the chance for a good outcome cannot be designed. Worse, if a patient is treated with chemotherapy or radiation therapy for the wrong diagnosis, there is a risk of exposing the patient to toxicity without a chance for cure. In the spectrum of cancer care, there are five components relevant to patient care, involving specimen procurement, processes, report delivery, and initiation of care (Fig 1).^7^ Although all of these components are critical to the timely and effective treatment of patients with cancer, we chose to focus on those under control of the treating physicians and pathology laboratory. Specifically, we measured time from biopsy to creation of the pathology report. Other factors can cause delay in diagnosis, including when a patient decides to seek care and time from initial clinical evaluation to performance of the biopsy. Once the clinician receives the results, they must still be transmitted to the patient, and a treatment plan must be developed and treatment initiated. In the best-case scenario, an optimized medical system can complete this process in a few days, and therapy can begin in a timely fashion. However, in many existing systems in LMICs, segments of this process can be greatly delayed for a broad range of reasons.^8^ Importantly, the first segment relies heavily on patient education and community engagement; however, the remaining steps fall to the medical system itself to improve and optimize, which includes development of a sustainable workforce, guaranteed supply chains for consumables, and modern systems for patient and laboratory information processing.^3,9^ In LMICs, diagnostic delays can have exponential implications because patients often initially present with advanced disease. Without optimized diagnostic and medical systems in place, patients will start their treatments with more-advanced disease and poorer prognoses than at initial presentation. Currently, there is limited turnaround time (TAT) data available from LMICs; however, typically TATs are longer than what is considered appropriate in high-income countries.^10^

As an integral part of the Butaro Cancer Center of Excellence (BCCOE), the pathology laboratory was opened in July 2012 and subsequently underwent an iterative development, progressively implementing key components of the process to enhance the cancer diagnostics of BCCOE. Before the opening of this laboratory, TAT in this setting could be measured in months because of inconsistent laboratory operations and a paucity of in-country pathologists.

The goal of this study was to determine the TATs for anatomic pathology specimens during the implementation and progressive development of our pathology laboratory at BCCOE in Rwanda.

## METHODS

### Setting

BCCOE is located in rural, northern Rwanda near the Ugandan border, managed by the Ministry of Health with the support of Partners In Health/Inshuti Mu Buzima, and provides clinical and hospital services to a catchment area of 336,455 people,^11^ as well as serving as a national referral center for cancer care. With the opening of this facility, a pathology laboratory was put in place, but there was no pathologist on site.^12^ From 2012 to 2017, this site evolved to now include full-service automated histology and immunohistochemistry (IHC) capabilities, an onsite pathologist, and static-image and whole-slide imaging telepathology systems connecting the site to 15 expert pathologists in the United States and Europe.

### Study Design

This was a retrospective study comparing TATs across three different diagnostic work streams that occurred during the pathology laboratory implementation at BCCOE. TAT for each diagnostic method was defined as the number of days between the date of the biopsy procedure and the date the finalized pathology report was generated (Fig 1). This study relied on data from 2013 through 2015, during the iterative development of diagnostic processes including the initial use of remote diagnostics and static-image telepathology and periodic onsite visiting pathologists.

It is important to note that these work streams do not represent a specific chronology or set duration of time. Instead, each work stream corresponds to a specific mode by which one can obtain a diagnosis, and work-stream development was iterative, not linear. Thus, the analyses are specific to each work stream or diagnostic mode rather than a particular time period. The diagnostic modes include:

#### Diagnostic mode 1.

Tissue blocks and slides were generated in Butaro and then physically transported to Brigham and Women’s Hospital (BWH) in Boston, Massachusetts, for evaluation and diagnosis.

#### Diagnostic mode 2.

Static images of slides prepared in Butaro by histotechnicians were uploaded to case-sharing software/platform (iPath; https://www.ncbi.nlm.nih.gov/pubmed/16375782) and evaluated and diagnosed remotely by pathologists at BWH.

#### Diagnostic mode 3.

Pathologists diagnosed patients on site at BCCOE through periodic visiting pathologists from the United States and Kigali.

Some samples were originally read through iPath, but because of a lack of diagnostic certainty, they were shipped to BWH for an additional evaluation. These patient cases were considered to be part of mode 1 and deleted from the mode 2 data. Outliers were defined as any TAT longer than 1 year and any mode 1 TAT shorter than 5 days; these were excluded from further analysis. 

As part of this iterative implementation approach, IHC, which is required for certain final diagnoses, was implemented in Butaro in 2014 and was made available throughout each diagnostic mode, occurring in either Boston or Butaro.

### Data Collection and Analysis

Data were compiled from BCCOE and BWH to create one data set of all patient cases from January 1, 2013, to December 31, 2015. Samples from 2012 were excluded because of inconsistent recordkeeping during the early operation of BCCOE. Only samples from biopsies performed at BCCOE were included in analysis. Samples that were received as fresh tissue, fixed tissue, or blocks/slides from other Rwandan institutions were excluded because of the uncertainty of events and timeline before arrival at BCCOE.

Statistical analysis was conducted with Microsoft Excel and STATA software (version 13; College Station, TX). Individual TAT was calculated for each patient sample, followed by the median TAT and interquartile range (IQR) for each year and diagnostic mode. 

A Kruskal-Wallis test was conducted to compare and assess statistical difference among the TATs across all three modes. A nonparametric test for trend was performed to test for a trend among the three diagnostic modes.

### Ethical Considerations

This study was approved by the Rwandan National Ethics Committee, BWH Institutional Review Board, and Inshuti Mu Buzima Research Committee.

## RESULTS

We originally identified 3,725 individual patient samples across the three phases of study and removed 211 (57 duplicates, 139 with either a missing biopsy or diagnosis date, and 15 outliers, all from mode 1); the final number of samples included in the analysis was 3,514. During the given time period, diagnostic mode 1 accounted for 2,695 patient samples, diagnostic mode 2 for 279, and diagnostic mode 3 for 540 (Table 1). Mode 1 had the highest total median TAT at 30 days (IQR, 22 to 43 days). For mode 2, the median TAT was 14 days (IQR, 7 to 27 days), and for mode 3, it was 5 days (IQR, 2 to 9 days; Table 1).

**Table 1:**
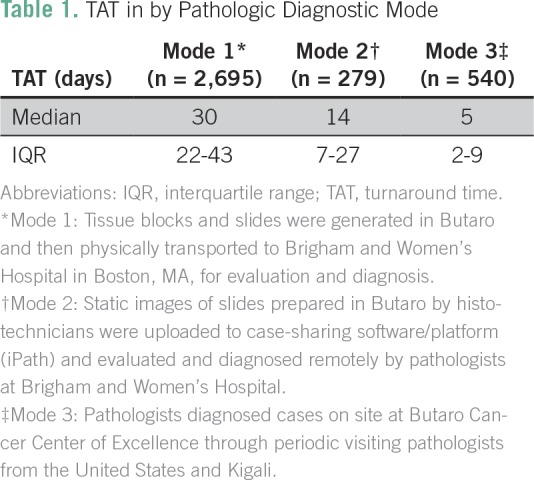
TAT in by Pathologic Diagnostic Mode

A Kruskal-Wallis test showed a statistically significant difference in median TATs across the three diagnostic modes (χ^2^ with ties = 1,378.78; *P* < .001). A nonparametric test for trend was significant (*P* < .001), demonstrating a significant decreasing trend in TAT across the three diagnostic modes (Table 2).

**Table 2:**
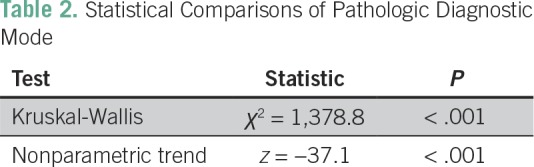
Statistical Comparisons of Pathologic Diagnostic Mode

## DISCUSSION

Ensuring accurate and timely pathology diagnoses is an essential component to cancer care in LMICs and remains an implementation challenge. Our experience demonstrates that developing pathology services is an iterative and ongoing process. Through evolving stepwise implementation using the diagnostic modes noted here, pathology diagnostic capacity on site in LMICs can sustainably increase, while simultaneously decreasing TAT from obtaining of a biopsy to diagnosis.

In 2012, trained histotechnicians initiated pathology services at BCCOE, such that tissue blocks and hematoxylin and eosin–stained slides were produced in a timely fashion. Diagnostic mode 1 was used in the absence of an onsite pathologist as an interim step so as to have reliable pathology while waiting for the availability of a trained pathologist on site. The primary delays that increased TAT were moving the samples from Butaro to Boston. The delays in this process were inconsistent, and the logistic challenges of this movement preceded the samples being received in Boston, where subsequently they were processed the same as routine BWH samples (data not shown). At the time, there were no neighboring countries with reliable, time-efficient pathology that had the capacity to accept the number of samples produced at BCCOE, and transport of material to either neighboring countries or the United States by mail or special carrier was unreliable. Therefore, samples were often carried to BWH in the United States by BCCOE travelers to assure their safe arrival. It was also not clear that samples could be shipped more quickly to a neighboring country than to the United States.

Elimination of these logistic challenges through diagnostic modes 2 and 3 has had the most impact on TAT. Investments in shipment demonstrated a more consistent means for transport and reception of samples; samples were delivered via weekly air shipments to Boston rather than carried by ad hoc travelers. Despite these improvements, delays in country were spread across technical and professional areas but were more consistent than those in diagnostic mode 1. Overall, we would recommend moving to complete in-country processing as quickly as possible during implementation because of the logistic challenges of moving samples from a location like Rwanda to a developed country like the United States. 

Informal discussions among clinicians at BCCOE have emphasized the need for as short a TAT as possible. Urgency of treatment initiation varies by disease, but all patients with cancer should start treatment as soon as possible for both physical and emotional reasons. Some circumstances, such as mediastinal lymphoma with compromise of chest structures and vasculature, are near emergencies regarding accurate diagnosis and treatment initiation. In general, a TAT of 5 days meets the needs of most patients; however, reducing this to 3 days, a common standard in the United States, would be ideal.

In LMICs, many pathology laboratories are staffed solely with histotechnicians; trained onsite pathologists may be absent or overburdened. It will also be years before there are an adequate number of trained pathologists in many countries in sub-Saharan Africa and similar sites. In an effort to fill this void, visiting short-term pathologists from abroad staffed our laboratory periodically. This did not replace the need for full-time pathologists but instead, taken together, demonstrated a dramatic effect on the rate of diagnoses. However, this was not a consistent approach, because pathologists were only on site intermittently. In addition, part-time Rwandan pathologists were hired to diagnose patients on site in Butaro. Although evaluation of patient cases was able to occur on site, this did not provide enough capacity to provide timely and accurate diagnoses for all patients.

Static-image telepathology (diagnostic mode 2), however, allowed for remote diagnoses to occur before pathologists were available full time on site. In the absence of a pathologist, technical staff were trained and outfitted with a photography protocol and system for collecting results using this static-imaging system. Implementation of this telepathology triage system was brought online in a phased approach to both train staff and optimize workflow from image upload to reception of results. Static-image diagnoses were compiled and compared with those made based on standard glass-slide histology, demonstrating a greater than 95% concordance rate.^13^ Although it is a more consistent method and a means of bridging the human resource gap, static-image telepathology was initiated at BCCOE only as an interim supplemental step. A full-time pathologist was hired in 2016, and additional analysis of TAT will be needed to fully understand the impact of this.

The data presented here do not include a breakdown of diagnosis by disease, but a majority of these patient cases were breast cancer. IHC is critical to many diagnoses and is essential to subsequent treatment decisions for patients with breast cancer. Determination of hormone receptor status of patients with breast cancer directly affects treatment choice and was part of the first set of IHC tests implemented on site at BCCOE. Before bringing IHC on site (now read via either telepathology or onsite pathology), all patient cases requiring IHC had to be sent to Boston. Incidence of breast cancer in LMICs is high, and additional analysis will be needed to better understand the impact of IHC on TAT for these patients. IHC was implemented at BCCOE in 2014; however, full analyses have not yet been performed to understand its overall impact on TAT for this factor alone. In 2016, fully automated histology and a whole-slide imaging system for enhanced telepathology were also installed, and their impacts are being evaluated, as are cost implications.

It is important to note that only developments in the preanalytic, analytic, and postanalytic phases of the pathology process are examined here. It is also critical for efficient and timely evaluation and scheduling of tissue acquisition to be routine in these environments, which is often not the case in Rwanda or elsewhere, and for initiation of treatment after the pathology report is received by the clinician to be efficient as well.

We believe the stepwise implementation of the pathology laboratory at BCCOE offers a sustainable model for scaling up capacity where both human and laboratory resources are constrained. Going forward, it will be critical to continue to measure TAT to assure sustainability and to explore more ways to improve pathology processes.
